# Diagnostic performance of an artificial intelligence algorithm for detecting pneumoperitoneum on abdominal CT scans

**DOI:** 10.1186/s13244-026-02348-8

**Published:** 2026-07-18

**Authors:** Yuwan Hu, Zhigang Sun, Haoyu Li, Shuya Gao, Haoran Du, Tongyin Zhang, Sheng Xie, Peng Wu, Pengbo Jiang, Dijia Wu, Xuemeng Wu, Hongliang Sun

**Affiliations:** 1 Department of Radiology, China–Japan Friendship Hospital, Beijing, China; 2 Graduate School, Chinese Academy of Medical Science & Peking Union Medical College, Beijing, China; 3 Department of General Surgery, China–Japan Friendship Hospital, Beijing, China; 4 Peking University China–Japan Friendship School of Clinical Medicine, Beijing, China; 5 Shanghai United Imaging Intelligence Co., Ltd., Shanghai, China

**Keywords:** Artificial intelligence (AI), Pneumoperitoneum, Tomography (X-ray computed), Sensitivity and specificity

## Abstract

**Objectives:**

This study aims to evaluate the diagnostic performance of an artificial intelligence (AI) algorithm for detection, segmentation, and volumetric quantification of pneumoperitoneum on abdominal CT scans.

**Materials and methods:**

We developed and validated a deep learning-based model for automated pneumoperitoneum detection on CT. Multi-center CT imaging series from 2072 patients were collected and randomly divided into training and testing sets at an approximate 7:3 ratio. The external validation set included 214 emergency CT scans collected between April 2022 and December 2024. Diagnostic reports served as the reference standard. Primary outcome included the area under the curve (AUC), sensitivity, specificity, accuracy, positive predictive value (PPV), and negative predictive value (NPV). Quantitative agreement between AI and reference volumes was assessed using the intraclass correlation coefficient (ICC).

**Results:**

In the test set (*n* = 607), the model demonstrated excellent performance: sensitivity 91.4%, specificity 93.1%, and AUC 0.97 (95% CI: 0.95–0.99). In the external validation cohort (*n* = 214), the model maintained robust performance with sensitivity 84.3% (95% CI: 76.2–90.5%), specificity 89.6% (95% CI: 82.3–94.6%), accuracy 86.9% (81.6–91.2%), PPV 89.2% (95% CI: 81.8–94.3%), and NPV 84.8% (95% CI: 77.1–90.7%). After excluding cases with minimal free gas (<1 mL), the model’s sensitivity improved to 96%. AI-derived volumes showed strong agreement with the reference standard (ICC 0.996, 95% CI: 0.994–0.997).

**Conclusion:**

The AI model attained high diagnostic accuracy for pneumoperitoneum on abdominal CT scans, promising to expedite emergency workflows.

**Key Points:**

***Question*** *Reliable AI detection of pneumoperitoneum, particularly for small-volume free air, on emergency CT remains an unmet need for rapid and accurate emergency triage.*

***Findings*** *The AI model shows high sensitivity and specificity for clinically relevant pneumoperitoneum volumes, although trace-volume detection on CT scans remains challenging.*

***Critical relevance statement*** *This study evaluates the diagnostic performance and volume-dependent variability of an AI model for pneumoperitoneum detection on CT scans, with the potential to aid emergency radiology workflow prioritization and decision support, though prospective studies are needed to confirm clinical impact.*

**Graphical Abstract:**

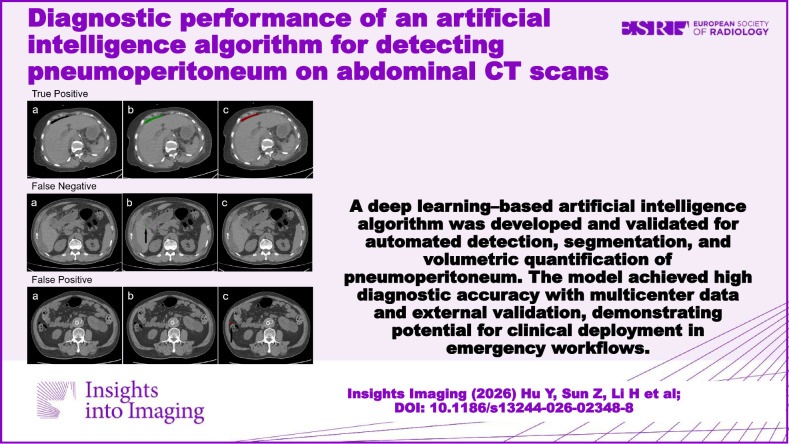

## Introduction

Pneumoperitoneum, defined as the pathological presence of air or gas within the peritoneal cavity, is the key indication of acute abdomen often indicating the presence of perforation, rupture and other serious lesions in the abdominal cavity [1]. Delayed diagnosis may precipitate severe complications such as peritonitis and sepsis, contributing to its alarmingly high 30-day mortality rate of up to 21% and substantially increased healthcare costs [2, 3]. Early detection is critical as it enables prompt surgical intervention, which is particularly vital for survival in patients with septic shock, thereby significantly improving clinical outcomes [4].

Current diagnostic modalities include abdominal radiography, ultrasonography, and computed tomography (CT), with CT serving as the diagnostic gold standard due to its superior sensitivity [5]. However, traditional manual interpretation CT image has time dependence and experience difference, which can easily lead to missed diagnosis or misdiagnosis. Diagnostic errors may directly impact patient management, with adverse outcomes reported in 7.2% of cases [6]. Recently, AI algorithm has made significant advancements in medical imaging technologies such as CT scans, X-rays, and ultrasonography [7–13], and deep learning algorithms hold potential in assisting the detection of pneumoperitoneum on CT scans [14, 15]. By enabling rapid and accurate analysis of large-scale imaging data, AI can reduce radiologist workload and improve diagnostic reliability, thereby addressing the limitations of traditional manual interpretation. In clinical practice, AI-based triage systems can be seamlessly integrated into radiology workflows through automated processing and real-time analysis. Results are typically presented as alerts or standalone interfaces alongside PACS, enabling rapid identification of high-risk cases. This approach shifts workflows from passive interpretation to proactive prioritization, reducing report turnaround time and facilitating earlier clinical intervention [16].

However, detecting trace pneumoperitoneum on CT remains inherently challenging due to its subtle appearance, small volume, and overlap with intraluminal gas. Prior studies have shown that AI performance is volume-dependent, with reduced sensitivity for minimal free air [14], highlighting an important limitation in current systems.

In this context, this study developed an AI algorithm for automatic detection, segmentation, and volumetric quantification of pneumoperitoneum on abdominal CT scans. The model employs a convolutional neural network (CNN) architecture designed to capture spatial and contextual features across both axial and longitudinal planes, enabling simultaneous precise segmentation and classification. This integrated approach generates both pixel-level visualizations and patient-level predictions. Compared with prior studies, the model adopts a three-dimensional (3D) architecture that captures spatial continuity and contextual information, which is critical for distinguishing free intraperitoneal gas from intraluminal bowel gas. A cascaded design incorporating anatomy-aware postprocessing was implemented to effectively suppress false positives. The system was rigorously validated using an external test set, suggesting robust performance and potential clinical applicability.

## Material and methods

Detection of pneumoperitoneum on CT imaging relies on identifying, localizing, and quantifying signs of free gas. Free gas within CT images exhibits extremely low Hounsfield Units (HU) values and manifests in variable morphologies and sizes, ranging from aggregated substantial collections to scattered minute bubbles. Given the frequent potential for confounding between free gas and pulmonary air or intraluminal gas within abdominal viscera (e.g., stomach and bowel), coupled with the inherent challenge of detecting small gas bubbles, we developed an AI algorithm for pneumoperitoneum detection on CT imaging. This algorithm was trained utilizing a substantial dataset annotated with gold standard labels.

This study represents a multi-center, retrospective investigation evaluating diagnostic test performance and was approved by the Institutional Review Board of China-Japan Friendship Hospital (CJFH) (approval no. 2023-KY-013-1). The requirement for informed consent was waived by the Institutional Review Board, as it used fully anonymized imaging data without clinical identifiers. After the test phase, data and model predictions were retrieved from the research database for further analysis.

The study comprised four phases: development data collection (Aug 2023–Feb 2024), model development and iteration (Sep 2023–May 2024), internal testing (Mar–May 2024), and external validation (Dec 2024–Jan 2025). The external validation cohort was temporally and institutionally independent from the development and internal datasets.

### Data distribution

We established a multi-center cohort for algorithm development, comprising 2072 patients with CT data collected from 31 clinical centers. Each patient corresponded to a single CT examination, resulting in 2072 volumetric CT datasets (i.e., full 3D volumes with dimensions of V × M × N). The scans were acquired from six major CT manufacturers (UIH, GE, Toshiba, Siemens, Philips, and NMS), as well as an additional unknown vendor (designated as ANO), and covered a wide range of slice thicknesses (0.3–5.0 mm) with full abdominal coverage (Supplement A).

Patient-level data splitting was performed at a 7:3 ratio, yielding a training cohort of 1465 patients (365 positive and 1100 negative cases) and an internal test cohort of 607 patients (140 positive and 467 negative cases). To prevent data leakage, splitting was strictly conducted at the patient level, ensuring that all axial slices from a given CT volume were exclusively assigned to either the training or test set, with no overlap between cohorts (Supplement B). At the patient level, diagnostic reports served as the gold standard reference. At the free gas level, all instances of free gas within the positive data were meticulously delineated by three annotators, and these annotations were subsequently reviewed and approved by a senior radiologist.

### Algorithm description

**Fig. 1:**
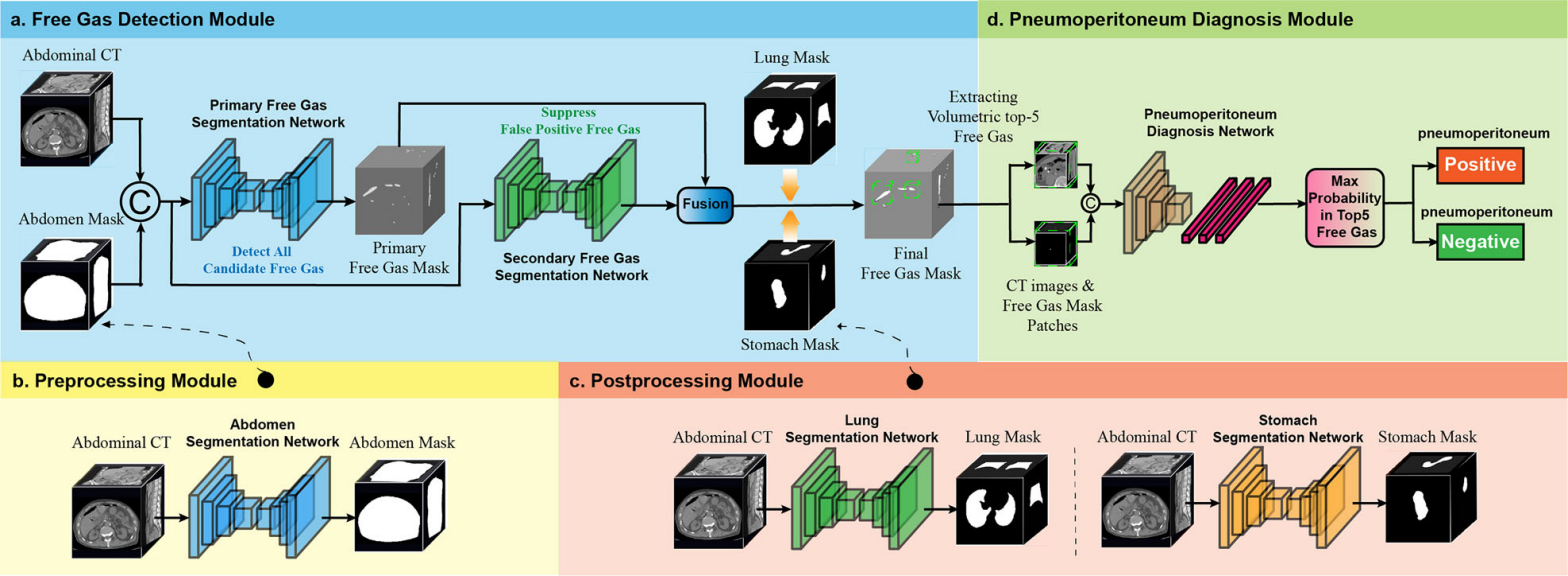
The pipeline of our CT-based pneumoperitoneum detection algorithm, including four major modules: **a** free gas detection module; **b** preprocessing module; **c** postprocessing module; and **d** pneumoperitoneum diagnosis module. **a** In this phase, the original abdominal CT image is cropped to a region of interest (ROI) defined by a bounding box derived from an abdomen segmentation mask. Due to anatomical variability across subjects, the size of the ROI varies. For instance, an original CT volume of size 350 × 350 × 610 (at 1 mm³ resolution) may be cropped to 281 × 213 × 391. Regardless of the initial ROI dimensions, each volume is uniformly patch-based resampled to a fixed input size of 160 × 160 × 160 before being fed into the segmentation network

Our CT-based pneumoperitoneum detection algorithm comprises four integrated modules: Preprocessing, Free Gas Detection, Postprocessing, and Pneumoperitoneum Diagnosis, illustrated in Fig. 1.

#### Preprocessing module

In Fig. 1b, An abdominal segmentation model extracts an abdominal region mask. The input image domain for subsequent free gas detection is confined to the abdominal bounding box defined by this mask.

#### Free gas detection module

**Fig. 2:**
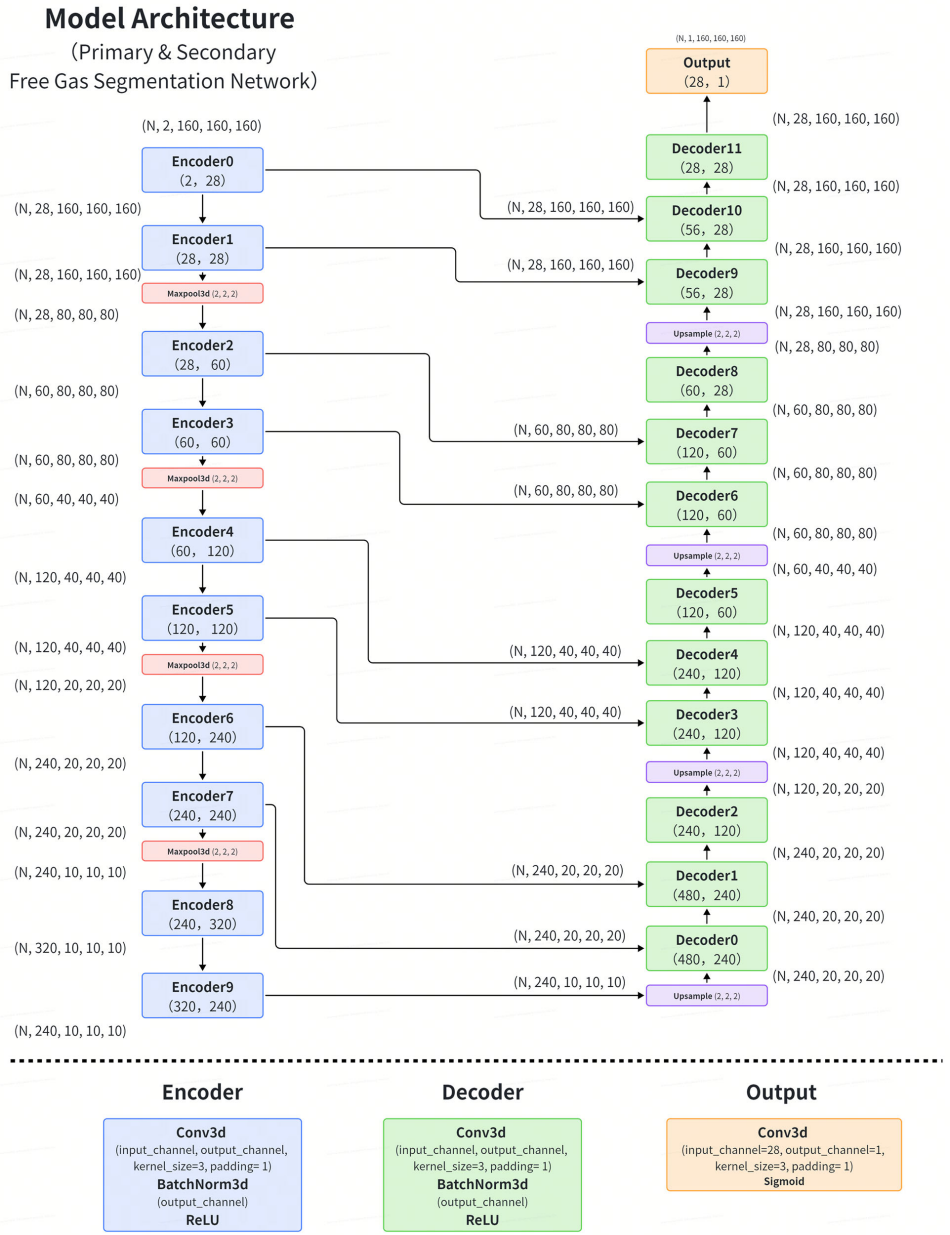
Model architecture of primary and secondary free gas segmentation network. The two free gas segmentation models (primary and secondary) share the same architecture. This structure is a variant of U-Net, consisting of symmetric Encoder and Decoder stages forming a 10-layer network (Encoder0–Encoder9, Decoder0–Decoder9). The Decoder stage additionally includes two extra layers (Decoder10 and Decoder11) to enhance network complexity. The final output of the Decoder stage is processed by an Output module to generate the probabilistic segmentation map of free gas. Both Encoder and Decoder modules share an identical structure, each composed of Conv3d (kernel size = 3, padding = 1), BatchNorm3d, and ReLU activation. The Output module contains a Conv3d layer (kernel size = 3, padding = 1) followed by a Sigmoid activation. The Encoder stage undergoes four MaxPooling3d operations for 1/2 down-sampling, while the Decoder stage uses four Up-sample operations to restore the original resolution. The numbers below each block (e.g., (60, 120)) denote the input and output channel dimensions. The size of input and output data for each layer are also indicated beside the corresponding blocks. The network adopts the hierarchical down-/upsampling and skip-connections of the U-Net family but includes several adaptations: (1) all convolutions are 3D; (2) downsampling uses MaxPool3D; (3) encoder and decoder pathways are deepened to 10 layers each; (4) BatchNorm3D stabilizes training; and (5) skip-connections are selectively applied rather than fully connecting all layers

In Fig. 1a, this module employs two cascaded segmentation networks for localizing all potential gas instances at the free gas level. Both the primary and secondary segmentation networks utilize identical architectures: a 4-layer symmetric encoder-decoder structure. Inputs consist of dual-channel images (abdominal-restricted CT and abdominal mask). During the encoder phase, features are progressively extracted via pooling layers. The decoder phase progressively restores the spatial resolution. Both phases are constructed using fundamental components including 3D convolutional layers (Conv3D), 3D batch normalization (BatchNorm3D), and ReLU activation function. A final sigmoid activation layer outputs a probability map of free gas. Following prior lesion detection studies such as CT pulmonary nodule detection [17], the primary segmentation network detects all candidate free gas regions. The secondary network then suppresses false positive gas regions identified by the primary network. This strategy reduces false positives with minimal impact on recall (Supplement C), which is clinically justified since a single true lesion confirms diagnosis, whereas false positives may lead to misdiagnosis. The two free gas segmentation models are illustrated in Fig. 2.

#### Postprocessing module

In Fig. 1c, this stage incorporates dedicated segmentation networks for the lungs and stomach. Based on the resultant lung and stomach masks, all detected free gas instances undergo processing to filter out apparent (“false”) free gas residing within pulmonary or gastric compartments, thereby further reducing false positives.

#### Pneumoperitoneum diagnosis module

**Fig. 3:**
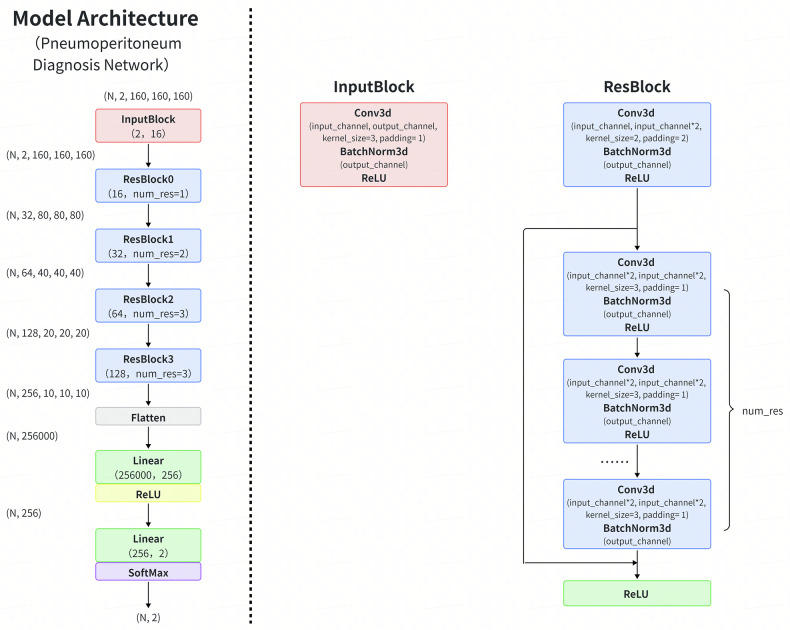
Model architecture of pneumoperitoneum diagnosis network. The model is designed based on residual connections. It begins with an InputBlock containing Conv3d (kernel size = 3, padding = 1), BatchNorm3d, and ReLU. This is followed by four consecutive ResBlocks. Each ResBlock first applies a down-sampling module (Conv3d with kernel size = 2, padding = 2, BatchNorm3d, and ReLU) for 2× reduction, followed by num_res residual modules (each consisting of Conv3d, BatchNorm3d, and ReLU, except the last module which omits ReLU). The residual sum is activated by ReLU. The output of the four ResBlocks is flattened into a 1D vector, processed by two Linear layers, and finally activated by SoftMax for classification probability. The numbers below InputBlock and Linear blocks represent input/output channels, while those below ResBlocks indicate input channels and the number of residual modules. Data dimensions for each layer are annotated adjacent to the blocks

In Fig. 1d, this module determines the presence of pneumoperitoneum at the patient level, based on the processed free gas detection results. It first extracts the volumetric top-5 candidate gas instances. Each instance is fed into a classification network to compute its diagnostic probability. The classification decision for pneumoperitoneum is then made based on the instance among the top 5 exhibiting the highest probability score. Inputs to this network are dual-channel patches (CT image and mask) centered on the candidate gas instance. Feature extraction involves downsampling via a 4-layer convolutional network, followed by two fully connected layers, culminating in a softmax activation function that outputs the final diagnostic probability. The detailed architecture of the pneumoperitoneum diagnosis model is shown in Fig. 3.

### Training strategy

The training of both segmentation networks within the free gas detection module was guided by a combined loss function incorporating Dice loss and focal loss [18], optimized using the Adam optimizer. Critically, for the primary segmentation network (aimed at identifying all possible gas), the loss calculation encompassed the entire abdominal region. Conversely, for the secondary network (focused on false positive reduction), the loss calculation was constrained solely to the regions identified as free gas by the primary network. Native CT volumes underwent resampling to isotropic [1 mm, 1 mm, 1 mm] voxels and were partitioned into [160, 160, 160] patches. Network training proceeded with a batch size of 8 on two NVIDIA A100 GPUs. Additionally, the grayscale intensity distribution of the input images was normalized to a window width (WW) of 500 HU and window level (WL) of 50 HU, optimized for visualization of abdominal free gas. The classification network within the Pneumoperitoneum Diagnosis Module was trained using cross-entropy loss, also optimized by Adam. All network training incorporated data augmentation techniques, including random translation, rotation, and scaling, to enhance model robustness. The learning rate and training epochs are summarized in Table 1.

**Table 1:** Training hyperparameters for primary and secondary free gas segmentation network and Pneumoperitoneum diagnosis network

	Loss function	Training epoch	Learning rate	Batch size	Optimizer	Input image size
Primary free gas segmentation network	λ Dice loss + (1 − λ) Focal loss	500	0.0001	8	Adam	160 × 160 × 160 (spacing 1 mm × 1 mm × 1 mm)
Secondary free gas segmentation network	λDice loss + (1− λ) Focal loss	500	0.0001	8	Adam	160 × 160 × 160 (spacing 1 mm × 1 mm × 1 mm)
Pneumoperitoneum diagnosis network	Cross-entropy loss	1000	0.0002	16	Adam	160 × 160 × 160 (spacing 1 mm × 1 mm × 1 mm)

^*^ λ for fusing Dice Loss and Focal Loss was set as 0.3

^*^ Parameters for Adam optimizer was {“betas”: (0.9, 0.999), “eps”: 1e-8, “weight_decay”: 1e-4, “amsgrad”: false}

### External validation

**Fig. 4:**
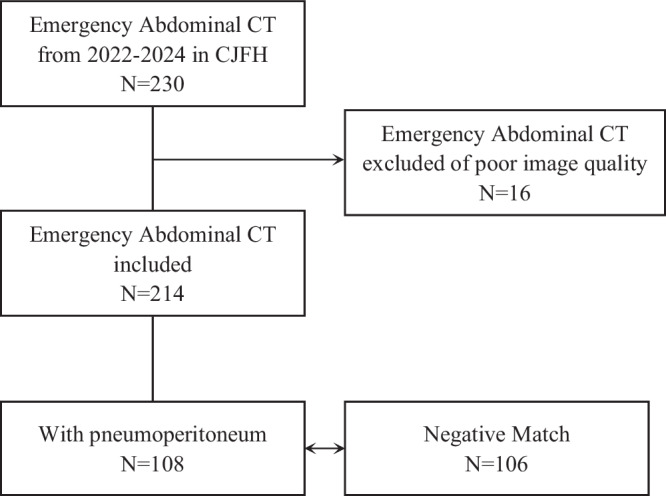
Inclusion flowchart of the study’s external validation cohort. ‘*N*’ indicates the number of CT examinations at each step. CJFH, China–Japan Friendship Hospital

This retrospective diagnostic performance study utilized an independent validation cohort of 214 emergency abdominal CT scans (108 positive, 106 negative) acquired at CJFH between April 2022 and December 2024. All consecutive scans during this period were systematically collected alongside corresponding radiological reports. Inclusion was limited to noncontrast axial CT images with a standardized 5-mm slice thickness and complete abdominal coverage. Scans with technical errors, significant artifacts, or missing reports were excluded. The patient selection and analysis workflow is illustrated in Fig. 4.

#### Pneumoperitoneum compartmentalization

We conducted a systematic evaluation of intraperitoneal free gas presence and spatial distribution using a standardized five-region CT assessment protocol. This methodical approach encompassed detailed analysis of five critical anatomical compartments: (1) the subphrenic space, (2) porta hepatis region, (3) mid-anterior abdominal wall space, (4) intermesenteric space, and (5) pelvic cavity. In region-based analyses, compartments were treated as non-mutually exclusive, and each region was evaluated independently. Sensitivity and specificity were calculated using region-specific reference standards, with positive cases defined as the presence of free gas within a given region and negative cases as its absence, regardless of involvement of other regions.

#### Pneumoperitoneum grading

Volumetric assessment of free intraperitoneal gas was performed on a Nebula Workstation (IntelliSpace Portal 10.1, Philips) using manual segmentation of axial CT images, with careful exclusion of intraluminal bowel gas. Thin-slice (5-mm) axial images with multiplanar reconstruction (MPR) were utilized in conjunction with a semi-automated volumetric tool to quantify total gas volume. For clinical correlation, the measured volumes were classified into four ordinal grades: (1) none (absence of detectable free air), (2) small amount (total volume < 2 mL), (3) moderate amount (2 < total volume < 50 mL), and (4) large amount (total volume > 50 mL).

### Reference test

The reference standard for the primary evaluations of the external validation cohort was established based on diagnostic reports. For subgroup analyses within the external validation, two fellowship-trained abdominal radiologists (H.S. and Y.H., with 25 and 5 years of post-fellowship experience, respectively) independently reviewed all CT scans under partially blinded conditions. Specifically, they had access to basic clinical information but remained blinded to the radiology reports and AI model outputs. Their systematic analysis included assessing the spatial distribution and volumetric quantification of free intraperitoneal air. Discordant cases were resolved through consensus review.

### Statistics

The study aimed to evaluate the diagnostic performance of the AI model in diagnosing pneumoperitoneum on abdominal CT scans, with continuous variables reported as means and standard deviations (SD), and categorical variables as counts and percentages. Interobserver agreement for volumetric measurements was assessed using intraclass correlation coefficient (ICC). Agreement between AI-derived measurements and the reference standard was comprehensively evaluated using volumetric correlation analysis and ICC. Model performance was assessed on both training and test sets. The primary outcome was the area under the curve (AUC) compared with reference standard. Additional metrics—including sensitivity, specificity, accuracy, positive predictive value (PPV), and negative predictive value (NPV)—were calculated with 95% confidence intervals. External validation was performed using an independent test set. Subgroup analyses were conducted to evaluate performance across anatomical locations (e.g., subphrenic, pelvic), etiologies (e.g., perforation, trauma, and postoperative), and pneumoperitoneum volume categories. All statistical calculations were performed using SPSS 26.0 (IBM Corp.).

## Results

### Data set characteristics

A multicenter dataset of 2072 abdominal CT scans was used for model development and validation. The baseline characteristics are presented in Table 2. The training set included 365 pneumoperitoneum-positive (mean age 59 ± 19.3; 34.2% female) and 1100 negative cases (mean age 57 ± 17.9; 44.8% female). The test set contained 140 positive (mean age 60 ± 19.0; 37.1% female) and 467 negative cases (mean age 55 ± 16.6; 44.3% female).

**Table 2:** Data set characteristics

Patient characteristics	Training set value (SD)/*N* (%)		Test set value (SD)/*N* (%)		External validation set value (SD)/*N* (%)	
	Positive	Negative	Positive	Negative	Positive	Negative
Total CT scans	365	1100	140	467	108	106
Age	59 (19.3)	57 (17.9)	60 (19.0)	55 (16.6)	59 (17.1)	46 (17.8)
Female	125 (34.2%)	493 (44.8%)	52 (37.1%)	207 (44.3%)	40 (37.0%)	51 (48.1%)

*CT* computed tomography, *SD* standard deviations

### Threshold selection strategy

The optimal diagnostic threshold for pneumoperitoneum was determined using the internal test cohort (*n* = 607) without training data leakage. Six candidate thresholds (0.5–0.95) were evaluated (Supplement D). A threshold of 0.7 was selected for its balanced performance—robust sensitivity and high precision, the latter being critical in low-prevalence settings to reduce false positives and avoid unnecessary invasive workup. Given that the same internal test cohort was used for both threshold selection and initial performance estimation, the reported sensitivity, specificity, and accuracy for this cohort should be interpreted as optimistically biased estimates. Therefore, the independent evaluation of the fixed threshold (0.7) was performed on the external validation cohort (*n* = 214), where no further adjustments were made. This external validation provides the primary evidence of model robustness and cross-center generalizability.

### AI algorithm performance

At the patient level, our AI pneumoperitoneum detection algorithm achieved 91.4% sensitivity, 93.1% specificity, and an AUC of 0.97 (95% CI: 0.95–0.99) on the prospective test set (*n* = 607). In practical deployment testing on a single NVIDIA A4000 graphics processing unit (GPU), the algorithm demonstrated an average execution time of 26.56 s per case, with a video random access memory (VRAM) utilization of approximately 5175 megabytes (MB) (Table 3).

**Table 3:** Overall performance of AI in test set

Performance metrics	Training set value (95% CI)	Test set value (95% CI)	External validation set value (95% CI)
Sensitivity (%)	98.9 (97.2–99.6)	91.4 (85.6–95.0)	84.3 (76.4–90.2)
Specificity (%)	99.6 (99.1–99.9)	93.1 (90.5–95.1)	89.6 (82.5–94.5)
Accuracy (%)	99.5 (98.9–99.7)	92.8 (90.4–94.6)	86.9 (81.6–91.2)
Positive predictive value (PPV) (%)	98.9 (97.2–99.6)	80.0 (73.1–85.5)	89.2 (81.8–94.2)
Negative predictive value (NPV) (%)	99.6 (99.1–99.9)	97.3 (95.4–98.5)	84.8 (77.1–90.5)

*CI* confidence limits

### External validation

**Fig. 5:**
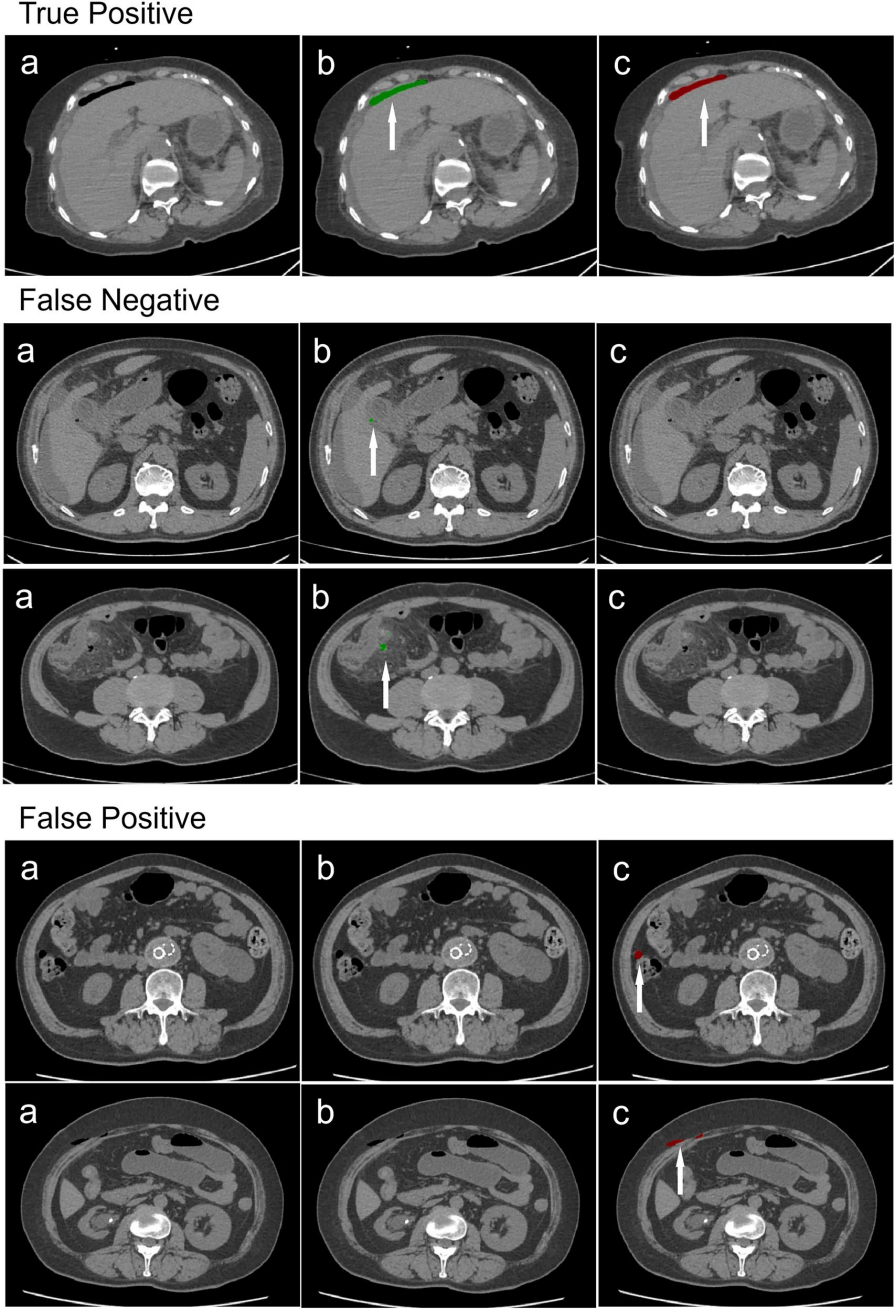
Representative cases illustrating model inference outcomes: true positive, false negative, and false positive. For each, **a** shows the original CT image, **b** the ground truth annotation (outlined in green), and **c** the model-predicted segmentation (outlined in red)

The external cohort from China-Japan Friendship Hospital comprised 214 patients: 108 pneumoperitoneum-positive (mean age 59 ± 17.1; 37.0% female) and 106 negative controls (mean age 46 ± 17.8; 48.1% female) (Table 2). The model achieved an overall sensitivity of 84.3% (95% CI: 76.4–90.2), specificity of 89.6% (95% CI: 82.5–94.5) and accuracy of 86.9% (95% CI: 81.6–91.2), with PPV of 89.2% (95% CI: 81.8–94.2) and NPV of 84.8% (95% CI: 77.1–90.5) (Table 3 and Fig. 5).

#### Stratified analysis by pneumoperitoneum regions and etiology

Diagnostic performance varied across anatomical regions (Table 4). The subphrenic space showed the best performance, with 100% sensitivity (95% CI: 92.3–100.0), 99.1% specificity (95% CI: 94.8–100.0), and 99.3% accuracy (95% CI: 96.2–100.0). The porta hepatis region also performed well with 92.7% sensitivity (95% CI: 80.1–98.5), 99.1% specificity (95% CI: 94.8–100.0) and 97.3% accuracy (95% CI: 93.1–99.3). Notably, the pelvic cavity and intermesenteric space showed relatively lower sensitivity (80.0%, 95% CI: 63.1–91.6 and 87.5%, 95% CI: 73.2–95.8, respectively), yet retained high specificity levels (100.0%, 95% CI: 96.6–100.0 and 95.3%, 95% CI: 89.4–98.5).

**Table 4:** AI model performance stratified by gas distribution and etiology

Free gas section	Number of cases (%)	Sensitivity (%)	Specificity (%)	Accuracy (%)	PPV (%)	NPV (%)
Subphrenic space	46 (42.6%)	100.0 (92.3–100.0)	99.1 (94.8–100.0)	99.3 (96.2–100.0)	97.9 (88.7–99.9)	100.0 (96.6–100.0)
Porta hepatis region	41 (38.0%)	92.7 (80.1–98.5)	99.1 (94.8–100.0)	97.3 (93.1–99.3)	97.4 (86.8–99.9)	97.2 (92.0–99.4)
Intermesenteric space	40 (37.0%)	87.5 (73.2–95.8)	95.3 (89.4–98.5)	93.2 (87.6–96.8)	87.5 (73.2–95.8)	95.3 (89.4–98.5)
Mid-anterior abdominal wall space	38 (35.2%)	92.1 (78.6–98.3)	96.2 (90.6–99.0)	95.1 (75.8–97.1)	89.7 (91.9–99.4)	97.1 (90.1–98.0)
Pelvic cavity	40 (37.0%)	80.0 (63.1–91.6)	100.0 (96.6–100.0)	94.5 (89.1–100.0)	100.0 (86.6–96.9)	93.0 (89.3–97.8)
Etiology						
Gastroduodenal	36 (33.3%)	91.7 (77.5–98.2)	98.1 (93.3–99.8)	96.5 (91.8–98.9)	94.3 (80.8–99.3)	97.2 (92.1–99.4)
Small bowel	21 (19.4%)	90.5 (69.6–98.8)	95.3 (89.3–98.5)	94.5 (89.3–98.5)	79.2 (57.8–92.9)	98.1 (93.0–99.8)
Colorectum	41 (38.0%)	73.2 (57.1–85.8)	96.2 (90.6–99.0)	89.8 (83.6–94.3)	88.2 (72.5–96.7)	90.3 (83.2–95.1)
Other (bladder)	1 (0.9%)	100.0 (20.7–100.0)	—	—	—	—
Trauma	2 (1.9%)	100.0 (34.2–100.0)	—	—	—	—
Post-operative	3 (2.8%)	100.0 (43.9–100.0)	—	—	—	—
Uncertain	4 (3.7%)	75.0 (43.9–100.0)	—	—	—	—

Etiology-based analysis revealed the best performance in gastroduodenal perforations, with 91.7% sensitivity (95% CI: 77.5–98.2), 98.1% specificity (95% CI: 93.3–99.8) and 96.5% accuracy (95% CI: 91.8–98.9). Small bowel perforations were detected with 90.5% sensitivity (95% CI: 69.6–98.8) and 95.3% specificity (95% CI: 89.3–98.5). Colorectal perforations showed comparatively lower sensitivity (73.2%, 95% CI: 57.1–85.8) but high specificity (96.2%, 95% CI: 90.6–99.0). In the traumatic (*n* = 2) and postoperative (*n* = 3) subgroups, the model achieved 100% sensitivity; however, the small sample size (5% of cases) limits interpretation (Table 4).

#### Stratified analysis by pneumoperitoneum volume

Interobserver agreement for volumetric measurements was excellent (ICC = 0.992; 95% CI: 0.988–0.994). There was excellent agreement between AI-derived and reference gas volumes, with Pearson correlation coefficient of 0.993 (*p* < 0.001) and ICC of 0.996 (95% CI: 0.994–0.997). Stratified analysis indicated generally consistent performance across volume ranges: ICCs were 0.904 (95% CI: 0.804–0.950) for < 2 mL, 0.962 (95% CI: 0.923–0.981) for 2–50 mL, and 0.993 (95% CI: 0.985–0.997) for > 50 mL.

Model sensitivity increased with pneumoperitoneum volume (Table 5). For small volumes (< 2 mL, 43.5% of cases), sensitivity was 68.1% with a 6.6% false positive rate. In moderate volumes (2–50 mL, 30.6%), sensitivity improved to 93.9%, with a false positive rate of 1.9%. For large volumes (> 50 mL, 25.9%), sensitivity reached 100%, with the false positive rate remaining at 1.9%. After excluding cases with < 1 mL of free air, sensitivity improved to 96% (67/70), and further to 97% (59/61) when restricted to cases with more than 2 mL, indicating a positive correlation between detection sensitivity and free air volume.

**Table 5:** AI model performance stratified by gas volume

Total volume of free air	Number of TP cases (%)	Sensitivity (%)	False positive rate (FPR) (%)
Total volume < 2 mL	47 (43.5%)	68.1 (32/47)	6.6 (7/106)
2 < Total volume < 50 mL	33 (30.6%)	93.9 (31/33)	1.9 (2/106)
Total volume > 50 mL	28 (25.9%)	100.0 (28/28)	1.9 (2/106)
Total volume > 1 mL	70 (64.8%)	96.0 (67/70)	—
Total volume > 2 mL	61 (56.5%)	97.0 (59/61)	—

## Discussion

Recently, AI has been increasingly applied to assist radiologists in image interpretation, particularly in X-ray imaging such as chest radiographs and fracture detection by reducing reading time and minimizing human error [8, 19]. Deep learning models achieve performance comparable to experienced radiologists in detecting pulmonary nodules and early lung cancer on CT, and can act as a “second reader” to improve detection rates [20]. In emergency settings, AI has been applied to detect acute conditions, including pulmonary embolism, intracranial hemorrhage [21, 22], and acute abdominal pathologies (e.g., appendicitis, bowel obstruction, and pneumoperitoneum), supporting lesion annotation, prioritization, and workflow optimization [23].

Notably, AI has demonstrated potential in detecting imaging features of intraperitoneal free gas, thereby supporting radiologic diagnosis and improving efficiency, particularly in emergency and nighttime settings [24]. Nonetheless, accurate CT-based detection remains challenging; while specificity is often high, sensitivity remains suboptimal [14, 25]. Previous models, predominantly based on two-dimensional (2D) segmentation, struggle to reliably distinguish free intraperitoneal gas from intraluminal bowel gas due to their morphological similarity [14]. 3D segmentation provides richer spatial and morphological information, improving characterization of gas distribution and differentiation from intestinal gas, with the potential to significantly increase detection accuracy in clinical practice [15]. In this study, we developed and validated a deep learning-based AI system for CT detection of free intraperitoneal gas designed to detect and localize free gas, followed by automated segmentation and volume quantification.

The proposed pneumoperitoneum detection algorithm comprises four integrated modules: preprocessing, free gas detection, postprocessing, and pneumoperitoneum diagnosis. The model employs a cascaded neural network architecture that performs automated abdominal cavity segmentation, dual-phase gas detection using complementary 3D networks, anatomy-aware false positive reduction via dedicated organ segmentation, and clinically interpretable probabilistic diagnosis. The slight performance decline in the external validation cohort may be attributed to several factors. First, the use of 5-mm thick-slice emergency abdominal CT scans from routine clinical practice likely reduced sensitivity for detecting small or subtle gas collections due to lower spatial resolution. Second, domain shift between the development and external datasets—including differences in scanner vendors, acquisition protocols, and patient populations—may have affected generalizability. Third, the exclusive use of noncontrast CT may have further increased the difficulty of distinguishing free intraperitoneal gas from adjacent structures. The model demonstrates particular strength in identifying microbubbles and suppressing false positives, critical for minimizing alarm fatigue. Its modular cascaded design supports a balanced sensitivity–specificity trade-off.

Pneumoperitoneum most commonly results from hollow viscus perforation, particularly gastroduodenal ulcer perforation, while other causes include abdominal surgery, trauma, infection, paracentesis, and pneumatosis intestinalis [26]. Free intraperitoneal air distributes according to gravity and peritoneal anatomy, preferentially accumulating in the subphrenic space, with right-sided predominance due to guidance by peritoneal ligaments and the right paracolic gutter [27, 28]. Based on a retrospective analysis of multi-slice CT images, we propose a novel “five-region classification” of intraperitoneal free gas, dividing the peritoneal cavity into subphrenic, porta hepatis, mid-abdominal wall, intermesenteric, and pelvic regions to standardize detection and clinical evaluation. The AI system demonstrated region-dependent performance, with higher diagnostic accuracy in upper abdominal regions (subphrenic and porta hepatis), likely due to larger gas volumes, stable anatomy, minimal bowel gas interference, and characteristic gas morphology. In contrast, sensitivity was lower in mid-lower abdominal regions (intermesenteric and pelvic), possibly related to smaller gas volumes, inflammatory changes, and overlap with bowel gas [29].

Subgroup analysis showed that diagnostic performance varied by etiology. Upper gastrointestinal perforations predominantly involved subphrenic and porta hepatis gas, whereas colorectal perforations were more challenging, likely due to complex anatomy and abundant intraluminal gas. Subgroup trends were consistent with the PACT-3D framework [15]; however, these comparisons are indirect, as no head-to-head evaluation was performed. Larger multicenter studies are needed to validate generalizability across etiologies.

Retrospective analysis of misclassified cases revealed that false negatives were primarily associated with scattered free gas, particularly in mesenteric or retroperitoneal regions, where morphology closely resembled normal intraluminal gas. The model demonstrated limitations in detecting tiny gas bubbles, commonly seen in diverticulitis or appendiceal perforation. In contrast, larger cumulative gas volumes were accurately identified, often indicating surgical emergencies. Meanwhile, the model showed high specificity, helping to reduce false-positive diagnoses and mitigating potential diagnostic fatigue. Analysis of false-positive cases identified key confounding factors, including air-containing abscesses, subcutaneous emphysema, intraluminal bowel gas distension, air-fluid levels, and artifact from prosthetic materials. The findings of this study align with the observations made by I-Min Chiu and colleagues [15]. However, as no direct comparison was conducted on the same dataset, these observations should be interpreted as indirect.

Recent advances in artificial intelligence (AI) have improved the detection of intraperitoneal free gas; however, identifying minimal volumes (< 5 mL) remains challenging. Prior studies, including Chiu et al [15], have demonstrated volume-dependent sensitivity, ranging from 89–91% at 1 mL to 95–98% at 10 mL thresholds. Consistent with these findings, our model achieved 96% sensitivity for volumes above 1.0 mL and 97% for above 2.0 mL, comparing favorably with previously reported results. Notably, this study introduces precise volumetric quantification of free gas using a clinically validated AI system, offering a quantitative tool to support early diagnosis and disease monitoring. The model shows high agreement with the reference standard across volume ranges, though performance decreases for trace pneumoperitoneum (< 2 mL).

This study has several limitations. First, sensitivity was relatively lower for trace pneumoperitoneum compared with larger gas volumes, indicating a need to improve detection of small-volume free gas. Second, the prevalence of pneumoperitoneum in the external validation cohort was artificially balanced (approximately 50%), which may limit generalizability to routine clinical settings. Finally, athough the model may facilitate rapid detection and triage of pneumoperitoneum in emergency settings, its real-world utility remains to be established and its clinical impact should be considered hypothesis-generating. Future studies should include prospective reader studies and silent-mode deployment to evaluate effects on radiologist performance, workflow efficiency, and clinical decision-making.

In conclusion, this study demonstrates the feasibility of developing a deep-learning model for the precise identification of pneumoperitoneum on abdominal CT scans. The model exhibits strong internal performance and robust, albeit slightly reduced, external performance. It shows promising performance in detecting and quantifying intraperitoneal free gas, with high specificity and sensitivity for clinically relevant volumes, although trace-volume detection remains challenging. These features highlight its potential to facilitate rapid emergency decision-making and improve patient outcomes. However, prospective multi-site and workflow-impact validation are explicitly required prior to broad deployment.

## Supplementary information

ELECTRONIC SUPPLEMENTARY MATERIAL

## Data Availability

The datasets are available from the corresponding author with a reasonable request.

## Abbreviations

3D: Three-dimensional
AI: Artificial intelligence
AUC: Area under the curve
BatchNorm3D: 3D batch normalization
CJFH: China-Japan Friendship Hospital
Conv3D: 3D convolutional layers
CT: Computed tomography
GPU: Graphics processing unit
HU: Hounsfield Units
ICC: Intraclass correlation coefficient
NPV: Negative predictive value
PPV: Positive predictive value
SD: Standard deviations

## References

[1] Tanner TN, Hall BR, Oran J et al (2018) Pneumoperitoneum. Surg Clin North Am 98:915–932 30243453 10.1016/j.suc.2018.06.004

[2] Tau N, Cohen I, Barash Y, Klang E et al (2020) Free abdominal gas on computed tomography in the emergency department: aetiologies and association between amount of free gas and mortality. Ann R Coll Surg Engl 102:581–589 32233866 10.1308/rcsann.2020.0057 PMC7538726

[3] Tolstrup M-B, Watt SK, Gögenur I et al (2017) Morbidity and mortality rates after emergency abdominal surgery: an analysis of 4346 patients scheduled for emergency laparotomy or laparoscopy. Langenbecks Arch Surg 402:615–623 27502400 10.1007/s00423-016-1493-1

[4] Azuhata T, Kinoshita K, Kawano D, Komatsu T, Sakurai A, Chiba Y (2014) Time from admission to initiation of surgery for source control is a critical determinant of survival in patients with gastrointestinal perforation with associated septic shock. Crit Care 18R87 24886954 10.1186/cc13854 PMC4057117

[5] Larsen NE, Mikkelsen E, Knudsen AR, Larsen LP (2021) Low-dose CT for diagnosing intestinal obstruction and pneumoperitoneum; need for retakes and diagnostic accuracy. Acta Radiol Open 10:2058460121989313 33786202 10.1177/2058460121989313 PMC7958640

[6] Ruchman RB, Jaeger J, Wiggins 3rdEF, Seinfeld S, Thakral V, Bolla S (2007) Preliminary radiology resident interpretations versus final attending radiologist interpretations and the impact on patient care in a community hospital. AJR Am J Roentgenol 189:523–526 17715095 10.2214/AJR.07.2307

[7] Immonen E, Wong J, Nieminen M, Kekkonen L, Roine S, Törnroos S (2022) The use of deep learning towards dose optimization in low-dose computed tomography: a scoping review. Radiography (Lond) 28:208–214 34325998 10.1016/j.radi.2021.07.010

[8] Meedeniya D, Kumarasinghe H, Kolonne S, Fernando C, Díez IT, Marques G (2022) Chest X-ray analysis empowered with deep learning: a systematic review. Appl Soft Comput 126:109319 36034154 10.1016/j.asoc.2022.109319 PMC9393235

[9] Shen YT, Chen L, Yue WW, Xu HX (2021) Artificial intelligence in ultrasound. Eur J Radiol 139:109717 33962110 10.1016/j.ejrad.2021.109717

[10] Cheng CY, Chiu IM, Hsu MY, Pan HY, Tsai CM, Lin CR (2021) Deep learning assisted detection of abdominal free fluid in Morison’s pouch during focused assessment with sonography in trauma. Front Med (Lausanne) 8:707437 34631730 10.3389/fmed.2021.707437 PMC8494971

[11] Chiu IM, Lin CR, Yau FF, Cheng FJ, Pan HY, Lin XH (2023) Use of a deep-learning algorithm to guide novices in performing focused assessment with sonography in trauma. JAMA Netw Open 6:e235102 36976564 10.1001/jamanetworkopen.2023.5102 PMC10051044

[12] Lu CY, Wang YH, Chen HL, Goh YX, Chiu IM, Hou YY (2025) Artificial intelligence application in skull bone fracture with segmentation approach. J Imaging Inf Med 38:31–46 10.1007/s10278-024-01156-0 PMC11811319 38954293

[13] Chiu IM, Chen TY, Zheng YC, Lin XH, Cheng FJ, Ouyang D (2024) Prospective clinical evaluation of deep learning for ultrasonographic screening of abdominal aortic aneurysms. NPJ Digit Med 7:282 39406888 10.1038/s41746-024-01269-4 PMC11480325

[14] Brejnebøl MW, Nielsen YW, Taubmann O, Eibenberger E, Müller FC (2022) Artificial Intelligence based detection of pneumoperitoneum on CT scans in patients presenting with acute abdominal pain: a clinical diagnostic test accuracy study. Eur J Radiol 150:110216 35259709 10.1016/j.ejrad.2022.110216

[15] Chiu IM, Huang TY, Ouyang D, Lin WC, Pan YJ, Lu CY (2024) PACT-3D, a deep learning algorithm for pneumoperitoneum detection in abdominal CT scans. Nat Commun 15:9660 39511229 10.1038/s41467-024-54043-1 PMC11544264

[16] Wiklund P, Medson K (2023) Use of a deep learning algorithm for detection and triage of cancer-associated incidental pulmonary embolism. Radiol Artif Intell 5:e220286 38074784 10.1148/ryai.220286 PMC10698599

[17] Chen L, Gu D, Chen Y, Shao Y, Cao X, Liu G (2021) An artificial-intelligence lung imaging analysis system (ALIAS) for population-based nodule computing in CT scans. Comput Med Imaging Graph 89:101899 33761446 10.1016/j.compmedimag.2021.101899

[18] Lin TY, Goyal P, Girshick R, He K, Dollar P (2020) Focal loss for dense object detection. IEEE Trans Pattern Anal Mach Intell 42:318–327 30040631 10.1109/TPAMI.2018.2858826

[19] Guermazi A, Tannoury C, Kompel AJ, Murakami AM, Ducarouge A, Gillibert A (2022) Improving radiographic fracture recognition performance and efficiency using artificial intelligence. Radiology 302:627–636 34931859 10.1148/radiol.210937

[20] Khalaji A, Riahi F, Rafieezadeh D, Khademi F, Fesharaki S, Joni SS (2025) Artificial intelligence in automated detection of lung nodules: a narrative review. Int J Physiol Pathophysiol Pharm 17:45–51 10.62347/YHID9574 PMC12089837 40401117

[21] Weikert T, Winkel DJ, Bremerich J, Stieltjes B, Parmar V, Sauter AW (2020) Automated detection of pulmonary embolism in CT pulmonary angiograms using an AI-powered algorithm. Eur Radiol 30:6545–6553 32621243 10.1007/s00330-020-06998-0

[22] Ahmed AE, Aljohani WF, Abu Rukbah LK, Rajhi SA, Najmi NK, Zughlul MK et al (2025) The role of artificial intelligence in stroke imaging in emergency settings: a systematic review. Cureus 17:e93941 10.7759/cureus.93941 PMC12588366 41200652

[23] Yao J, Chu LC, Patlas M (2024) Applications of artificial intelligence in acute abdominal imaging. Can Assoc Radiol J 75:761–770 38715249 10.1177/08465371241250197

[24] Winkel DJ, Heye T, Weikert TJ, Boll DT, Stieltjes B (2019) Evaluation of an AI-based detection software for acute findings in abdominal computed tomography scans: toward an automated work list prioritization of routine CT examinations. Invest Radiol 54:55–59 10.1097/RLI.0000000000000509 30199417

[25] Taubmann O, Li J, Denzinger F, Eibenberger E, Müller FC, Brejnebøl MW (2020) Automatic detection of free intra-abdominal air in computed tomography. In: Medical image computing and computer assisted intervention—MICCAI 2020. Springer, Cham

[26] Pinto A, Miele V, Laura Schillirò M, Nasuto M, Chiaese V, Romano L (2016) Spectrum of signs of pneumoperitoneum. Semin Ultrasound CT MR 37:3–9 26827732 10.1053/j.sult.2015.10.008

[27] Kim SH, Shin SS, Jeong YY, Heo SH, Kim JW, Kang HK (2009) Gastrointestinal tract perforation: MDCT findings according to the perforation sites. Korean J Radiol 10:63–70 19182505 10.3348/kjr.2009.10.1.63 PMC2647165

[28] Montanarella M, Boldig K, Virarkar M, Kumar S, Elsherif S, Lall C (2023) Intraperitoneal anatomy with the aid of pathologic fluid and gas: an imaging pictorial review. J Clin Imaging Sci 13:13 37292244 10.25259/JCIS_29_2023 PMC10246409

[29] Thorisson A, Nikberg M, Torkzad MR, Laurell H, Smedh K, Chabok A (2020) Diagnostic accuracy of acute diverticulitis with unenhanced low-dose CT. BJS Open 4:659–665 32431087 10.1002/bjs5.50290 PMC7397358

