# Supplementary material for: Diagnostic performance of an artificial intelligence algorithm for detecting pneumoperitoneum on abdominal CT scans
Source: Insights Imaging. 2026 Jul 18;17:186. doi: 10.1186/s13244-026-02348-8 (PMC13380604; doi:10.1186/s13244-026-02348-8)
Supplement: Supplementary file 1 — ELECTRONIC SUPPLEMENTARY MATERIAL [file 13244_2026_2348_MOESM1_ESM.pdf]

Diagnostic performance of an artificial intelligence algorithm for detecting pneumoperitoneum on abdominal CT scans

Supplementary material

Supplement A

Table 1. Detailed data distribution for patient cohort for algorithm development from multi-center

Site Name	Positive	Negative
Shanghai Sixth People's Hospital Affiliated to Shanghai Jiao Tong University	210	382
United Imaging Healthcare	0	39
Lingang Campus of Shanghai Sixth People's Hospital Affiliated to Shanghai Jiao Tong University	183	17
University Hospital of Tübingen	0	150
Fengjie County People's Hospital of Chongqing	24	4
Shanghai First Maternity and Infant Hospital Affiliated to Tongji University	42	255
Sun Yat-sen University Cancer Center	3	50
Beijing Friendship Hospital Affiliated to Capital Medical University	15	152
Peking University Third Hospital	7	36
Shanghai Tongji Hospital Affiliated to Tongji University	3	37
The First Hospital of China Medical University	1	6
Tongji Hospital Affiliated to Tongji Medical College of Huazhong University of Science and Technology	11	193
Affiliated Hospital of Chengdu University	2	22
Wuhan Third Hospital	4	100
Shanghai Ninth People's Hospital Affiliated to Shanghai Jiao Tong University School of Medicine	0	9
The First Affiliated Hospital of Xi'an Jiaotong University	0	15
General Hospital of Eastern Theater Command, PLA	0	49
Chengdu Sixth People's Hospita	0	6
Mianyang Central Hospital	0	17
Zhejiang Provincial People's Hospital	0	4
Shanghai Meinian Onehealth Healthcare	0	2
Nanjing Drum Tower Hospital, The Affiliated Hospital of Nanjing University Medical School	0	2
Huangshi Central Hospital	0	5
People's Hospital of Guangxi Zhuang Autonomous Region	0	1

Beijing Tongren Hospital Affiliated to Capital Medical University	0	3
Qingpu Branch of Zhongshan Hospital Affiliated to Fudan University	0	1
Fudan University Shanghai Cancer Center	0	4
Shanghai Public Health Clinical Center	0	3
West China Hospital of Sichuan University	0	1
Shanghai General Hospital (Shanghai First People's Hospital)	0	1
Shanghai Jiading District Central Hospital	0	1

Table 2. Detailed data distribution for patient cohort for algorithm development from various CT manufacturers

CT Manufacture	POS	Neg
UIH	292	331
GE	46	119
TOSHIBA	95	287
SIEMENS	21	320
PHILIPS	44	371
NMS	3	0
ANO	4	139

Supplement B

Detailed breakdowns of the dataset used for developing CT-based pneumoperitoneum detection algorithm

	**Positive**	**Negative**
**Training cohort**	365 cases<br>(190,443 axial slices)	1100 cases<br>(627,840 axial slices)
**Testing cohort**	140 cases<br>(80,676 axial slices)	467 cases<br>(269,060 axial slices)

Supplement C

Free gas Recall and FPPV on lesion-level in training set and testing set, comparing primary & secondary Free Gas Segmentation Network

Dataset	Cascaded Network	Recall	FPPV
Training Set	Primary	0.853	5.66
	Secondary	0.846	3.31
Testing Set	Primary	0.804	6.44
	Secondary	0.789	4.52

Supplement D

Core diagnostic metrics for different threshold on internal test cohort

Threshold	Sensitivity	Specificity	Precision
0.5	0.921	0.919	0.772
0.6	0.921	0.923	0.782
**0.7**	**0.914**	**0.931**	**0.800**
0.8	0.900	0.936	0.808
0.9	0.893	0.946	0.833
0.95	0.879	0.949	0.837
